# The dual nature of cooperation and its influence on SME's innovativeness

**DOI:** 10.1016/j.heliyon.2023.e16824

**Published:** 2023-05-31

**Authors:** Maciej Zastempowski

**Affiliations:** Faculty of Economic Sciences and Management, Nicolaus Copernicus University, Gagarina 13A Street, 87-100 Toruń, Poland

**Keywords:** Cooperation, External relationship, Internal relationship, Innovativeness, SMEs, Personality

## Abstract

In this paper, the external and internal cooperation determinants of the four types of innovation – product, process, organisational and marketing – are studied from the perspective of small and medium-sized enterprises (SMEs). From a theoretical standpoint, taking into account the dual nature of cooperation, cooperation determinants can be divided into two groups: external - comprising triple helix entities: universities, governments and industry, and internal – comprising employee personality traits: decision-making autonomy, creativity, willingness to cooperate, openness to changes, risk-taking and social empathy. Additionally, three control variables were considered: age, size and sector of economic activity. The data examined comes from an empirical study of a randomly selected representative sample of 1286 SMEs in the Kuyavian-Pomeranian voivodeship, a region in central-northern Poland. The empirical research was carried out between June and September 2019 using the CAPI method. The multivariate probit regression model was used to analyse the obtained data. The results indicate that only two factors directly connected with the triple helix are common and significant determinants explaining all SME innovations. These are cooperation with public administration in the field of financial support, and cooperation with clients. In turn, significant variation was observed in terms of personality traits being an essential element of internal cooperation that may influence SME innovations. A positive impact on the probability of implementing three of the four types of innovation was observed for two personality traits, namely creativity and social empathy.

## 1 Introduction

Ever since Schumpeter [1] pointed out that the process of creative destruction, based on innovation, is essential for the development of capitalism, innovation has become an essential feature of entrepreneurship, and interest in it has increased significantly [2]. Stejskal et al. even suggest that nowadays, entrepreneurship and innovation are the fundamental drivers of economic growth, the creation of wealth in an economy, and an enterprise's long-term survival, profitability, and sustainable growth [3]. In turn, Walsh et al. point out that there is a certain global consensus that innovation, together with science and technology, plays a central role in achieving the Sustainable Development Goals of the 2030 Agenda for Sustainable Development [4] adopted by all United Nations Member States [5]. Consequently, as suggested by Hernández-Trasobares and Murillo-Luna, innovation has become increasingly important over the last few decades [6].

Small and medium-sized enterprises (SMEs) also play an essential role in modern economies. In 2020, there were over 21 million SMEs in the European Union, which accounted for 99.8% of all enterprises in the non-financial sector. SMEs created nearly 53% of the total value added and provided work to over 65% of employees [7]. SMEs also play an important role in Poland, creating 49.6% of GDP and employing 67.8% of all employees in the enterprise sector [8].

When integrating problems connected with the issues of innovation and SMEs, it can be seen that despite many years of scientific studies, there are still many research gaps. As Mendoza-Silva suggests, one of the gaps is the study of the relationship between different types of cooperation and their impact on a company's capacity to create innovations [9]. This is primarily due to the dual nature of cooperation, demonstrated in two perspectives, i.e. external and internal [10].

The external perspective focuses on cooperation between organisations. Its foundations can be identified in research on inter-organisational relations initiated by Coase [11], which led to the development of transaction cost economics, e.g. by Williamson [12,13]. As Hillebrand and Biemans point out, this theory focuses on the costs associated with transferring ownership between independent parties, distinguishing between different markets and hierarchies (i.e. organisations) [10]. Previous research has also indicated the existence of relatively stable, long-term and interdependent relationships between organisations, which led to the development of the theory of network structures [10,14,15]. Such networks may contain a variety of organisations, including, but not limited to, customers, suppliers or competitors with whom the organisation joins forces to achieve predetermined goals [16]. It is also worth looking at external cooperation from the perspective of innovation. Here, the relations between organisations in national [17,18] and sectoral innovation ecosystems [[19], [20], [21]], the creation of open innovations [[22], [23], [24], [25]] and, above all, access to external knowledge built on this basis, all seem particularly important [26]. Previous research also points to the critical role of many issues in this area, including the triple helix model of interactions between academia, industry and government [27], external knowledge [28], collaboration with partners [29,30], external network attributes [31], and orientation towards customers and competitors [29,32].

The internal cooperation perspective also covers several interesting research problems. A significant part of the literature relates to cooperation between separate business functions in the context of product development. It mainly deals with issues at the intersection of R&D and marketing, perceived as key in developing new products [33,34]. In turn, other authors analyse the relationship of cooperation between marketing and the manufacturing [35,36], engineering [37] and purchasing departments [38]. Another area of research is also multi-functional teams, including representatives of various business functions and their effectiveness [39]. Internal cooperation is also examined in the context of enterprises' effects [40], performance in terms of competitiveness [41], or relations with TQM systems [42]. It is also worth pointing to studies focusing on the resources embedded between individuals and their networks of relationships. Examples of the problems discussed here include, among others, internal social capital - the ties and relations with other people within the firm [43], external social capital - the ties and connections with various outside contacts [44], personality [45], and the relation between innovation capability and social capital and knowledge [9].

It is also worth emphasising that when researching various aspects of internal cooperation, the main concern is the problem of the individual and their multiple features that can stimulate or inhibit this relationship [46,47]. Mendoza-Silva points to another important research gap related to innovation and internal cooperation [9]. This is the question about the impact of the specific personality traits of a company's members, which affect internal cooperation, on results in the field of innovation [9]. Previous studies show several attractive employee personality traits that can affect internal cooperation, and which are examined in the context of innovation, e.g., decision-making autonomy [48,49], creativity [29,50], willingness to cooperate [31,51], openness to changes [50], risk-taking [48,52], and social empathy [53].

Combining both indicated research gaps, the article aims to examine the impact of external and internal cooperation on the innovativeness of SMEs. External cooperation is analysed from the perspective of the triple helix, while internal cooperation is analysed in terms of selected employee personality traits. Meanwhile, the innovativeness of SMEs, according to the 3rd edition of the Oslo Manual [54], is considered from the perspective of four types of innovation: product, process, organisational and marketing.

For this purpose, the empirical part of the research uses data from a study conducted in 2019 on a randomly selected representative sample of 1286 SMEs from the Kuyavian-Pomeranian Voivodeship, a region in central-northern Poland.

The paper is structured as follows: the second section provides a theoretical framework and proposes research hypotheses; the third section discusses the research design, showing the method of obtaining the data, the research sample, variables and multivariate probit regression; the fourth section presents the model estimation results and their discussion; finally, the fifth section provides the main conclusions.

## 2 Theoretical background

External cooperation is one of the primary sources of building a competitive advantage [55]. Triguero et al. indicated that collaboration is a crucial factor in innovation, making it faster and easier [56]. Ties with other organisations can also be an essential means of acquiring resources for relationship partners, and can contribute to different types of innovation [57]. Consequently, enterprises that want to succeed in the market increasingly seek partners with which they can collaborate effectively and gain a competitive advantage [58]. In recognising that inter-organisational relationships and external knowledge are essential for innovative activities [9], it should be emphasised that they depend on many factors.

To analyse them, it is worth focusing on the innovation ecosystem [17,18,20,21,59,60], which may have essential attributes significantly influencing firm innovation capabilities and performance. The literature defines the innovation ecosystem as a geographically limited system of various actors involved in innovation creation and diffusion [21]. As Breschi and Malerba note, companies, universities, professional schools, public research institutes, the government, and the links between them are most often mentioned in this case [20]. However, the innovativeness of these different types of cooperation (between various actors) may be different, as indicated by Giovannetti and Piga, who suggest that it depends, among others, on the forms of cooperation (tacit or explicit) and the use of ICT [61]. As pointed out by Audretsch and Belitski, an important role is also played by investment in R&D, knowledge spillovers, and innovation strategies [62]. In the context of cooperation in creating innovation, the concept of the triple helix, which is related to the ecosystem, is also essential [27]. Its authors, Etzkowitz and Leydesdorff, initially proposed this model to explain innovation as the result of collaborative creative processes involving and encompassing three main actors: universities, industry and governments [27,63]. However, it is worth emphasising that the innovation environment varies between regions and countries, and that distinct determinants act differently [64]. This difference is particularly visible between Western European countries and Central and Eastern European (CEE) countries [64,65]. Hudec even points out that many years after European Union integration, CEE countries still exhibit higher trust and limited cooperation between the triple helix entities - industry, public institutions and knowledge institutions [66]. On the other hand, a study by Hernández-Trasobares and Murillo-Luna in Spain confirmed the positive effect of cooperation on business innovation – the triple helix agents were considered necessary both individually and in various combinations [6].

In the case of cooperation with universities, research conducted so far shows various results. On the one hand, there is the aforementioned lack of trust and limited cooperation with universities in CEE countries [64], as well as the sometimes limited cooperation in Western European countries, e.g., in Belgium manufacturing [67]. On the other hand, the significant role of universities in innovation processes has been demonstrated in several studies. As knowledge is the foundation of innovation, Lehmann et al. suggest that the impact of universities, as an essential source of knowledge spillovers, is undisputed [68]. Maietta focused on analysing the drivers of R&D collaboration between universities and low-tech companies. The research results showed that geographical proximity to a university positively impacts product innovation, but negatively affects the amount of codified knowledge production [69]. Using 2012 Community Innovation Survey data, Prokop and Stejskal found that cooperation with universities or other higher education institutions affects the innovativeness of countries classified as low innovators, according to the Innovation Union Scoreboard. However, the same cooperation in the case of countries belonging to the group of moderate and strong innovators did not have a statistically significant impact [70]. Prokop et al. also pointed out that in the case of CEE countries, the transfer of knowledge and technology between academia and industry can stimulate innovation [64]. It is also worth noting that if we look at innovation from the perspective of patents (treated as one of the key measures of innovation outputs [9]), the role of universities as the source and basis of commercial technologies is powerful, as emphasised by Henderson et al. [71]. Consequently, the first hypothesis was proposed.

H1 Cooperation with a university positively affects SMEs' innovativeness.

As far as governments are concerned, such cooperation may boost firms' competitiveness by promoting innovation in private firms [6]. Research by Martínez-Román et al. indicated the impact of the backing received from public administration on the implementation of product and process innovations by Spanish SMEs [48]. Interestingly, the research results presented by Prokop et al. did not show that cooperation with the government influenced the innovativeness of CEE companies [64]. An exciting observation also comes from research by Bodas Freitas and Von Tunzelmann [72]. They suggested that government support for firms in the innovation adoption process can be provided in two ways: by offering financial support and by developing appropriate structures to offer technical consultancy, advice or information so as to support the diffusion of innovation. Accordingly, the following hypothesis was formulated.

H2 Cooperation with public administration positively affects SMEs' innovativeness.

In turn, in the field of cooperation between firms, e.g., joint R&D ventures, it should be emphasised that this can take different forms: collaboration with suppliers or clients (vertical), with competitors (horizontal), and with other enterprises within the enterprise group [2]. Cooperation with clients allows firms to both recognise and predict customer needs and opportunities [6,48,73], as well as reduce the risk related to innovations [48]. However, it is worth recalling the opinion of Christensen that a solid attachment to existing customers may lead to neglect of new niches appearing in the market [74]. Such niches can be built on disruptive innovation that creates a new market and value network, eventually displacing established market-leading firms, products and alliances. The critical role of customers as a source of innovation has already been pointed out by von Hippel, who studied the electronics industry and indicated that innovators are most often users [75]. In turn, other studies have argued that cooperation with customers impacts innovation output levels negatively [76]. Taking into account the above, another hypothesis was proposed.

H3 Cooperation with clients positively affects SMEs' innovativeness.

Cooperation with suppliers can bring a company various benefits, e.g., improving the quality of products and services or reducing costs due to business process innovation [48,73]. Collaborating with a supplier, as von Hippel suggests, can also lead to developing an innovation the company did not expect to use or sell if that innovation increases demand for something they want to sell [75]. On the other hand, Zhang and Merchant argue that the government can also play the supplier role as it is the dominant supplier of reliable institutional knowledge. Their research confirmed the role of cooperation with such a supplier in building the innovative capacity of enterprises [29]. Also, Akman and Yamliz pointed to the critical role of collaboration with suppliers in creating innovation capacity [77]. Consequently, the following hypothesis was proposed.

H4 Cooperation with suppliers positively affects SMEs' innovativeness.

Cooperation with competitors, in other words co-opetition, as indicated by Hernández-Trasobares and Murillo-Luna, is intended as a positive sum game in which both parties share the risks of developing technological innovations [6]. On the one hand, research by Giovannetti and Piga suggests that active cooperation in innovative activity between competitors lowers their innovation indicators and indirectly their productivity [78]. Iammarino et al. indicate that cooperative links with competitors do not seem to have a significant impact on increasing the technological capacity of companies [79], while Park and Lee show that effect of R&D cooperation with competitors on the firm's R&D intensity is negative [80]. On the other hand, co-opetition in innovation can bring the participating entities several benefits, e.g., access to knowledge and technical skills, the joint creation of knowledge, efficiency in the use of resources, or the exploration of new market opportunities [6,32,49,81]. The arguments presented above lead to the formulation of the following hypothesis.

H5 Cooperation with competitors positively affects SMEs' innovativeness.

Also, cooperation in innovation with other enterprises within the enterprise group can be practical. As suggested by Tether, firms which belong to the enterprise group are better informed of the capabilities of potential partners due to knowledge pooling and the activities of other members of their group [82]. Prokop et al. indicated that this kind of cooperation is also essential and significantly influences innovation outputs in all CEE countries except Slovakia [64]. Accordingly, the following hypothesis was formulated.

H6 Cooperation with other enterprises within the enterprise group positively affects SMEs' innovativeness.

Seen from the innovation perspective, internal cooperation is based primarily on the resources embedded among individuals (based on the resource-based view) [[83], [84], [85]], their networks of relationships [43,86], and the absorptive capacity [25,87] built as a result. Several studies indicate the critical role played here by internal social capital, the foundation of which is ties and relations with other people within the firm [43,88]. In turn, other studies highlight the essential function of personality factors in this area [45]. Personality is a concept many disciplines are interested in evaluating [89]. Personality traits perceived from the psychological perspective - the Big Five personality model [90] - has been the subject of several studies in the context of individual innovativeness [91], individual innovation behaviour in the workplace [92], innovativeness at the national level [93] or National Innovation Scores [94]. Studies by Mustafa et al. showed that openness to experience moderates the relationship between job satisfaction and innovative behaviour at work [95]. Saatci and Ovaci found that openness, conscientiousness, extraversion and neuroticism are relevant to individual innovation competencies [96]. Abdullah et al. showed that people with the personality traits of high extraversion and openness to experience are more creative than others [46]. On the other hand, Rodrigues and Rebelo suggested that an individual's disposition represents a valid and meaningful predictor of personal innovative performance [97].

It is worth agreeing with Mendoza-Silva that such personality traits are often analysed as an element of the inter-organisational characteristics of a firm's innovation capability [9]. Among employees' interesting personality traits that can affect internal cooperation and which are studied in the context of innovation, it is worth mentioning decision-making autonomy [48,49], creativity [29,50], willingness to cooperate [31,51], openness to changes [50], risk-taking [48,52] and social empathy [53].

Martínez-Román et al., examining the innovative capability of Spanish SMEs, suggested that managers' decision-making autonomy is essential for SMEs' innovativeness. In turn, the decision-making autonomy of non-managers had no such impact [48]. Similarly, Russell [98], as well as Hull and Covin [99], emphasised that the degree of decentralisation of decision-making is of great importance for the innovative activity of companies. Also, studies of enterprises in Andalusia have shown that the decision-making autonomy of managers is a factor affecting the introduction of both product and process innovations [49]. The role of autonomy in decision-making in building the innovative capability of Chinese enterprises was also noted by Yam et al. [100]. Consequently, the following hypothesis was proposed.

H7 Decision-making autonomy positively affects SMEs' innovativeness.

As Runco points out, creativity is an important part of cognitive, social and emotional activity [101]. It also plays a vital role in building innovation [29,50,52,102]. Its foundations are related to Guilford's theory of divergent and creative thinking [103]. Creativity is also often treated on an equal footing with innovation, which, as de Bess and Kotler suggest, is a mistake made by many companies [104]. Research by Martínez-Román et al. showed that creativity as an element of the promotion of managers and non-managers does not significantly affect the innovativeness of SMEs [48]. Zhang and Merchant suggest that creativity, as the ability to reconfigure a company's existing resources, is an essential element that creates the company's innovative capacity [29]. Mazzucchelli et al. point to the role of creativity in a team's innovative capacity [50]. Meanwhile, Lin, in a study on knowledge sharing and its impact on a company's ability to innovate, showed the vital role of creativity [52]. Dyer et al. have demonstrated that the ability to think creatively is also one of the critical traits that formulate the DNA of disruptive innovators [105]. Accordingly, the following hypothesis was formulated.

H8 Creativity positively affects SMEs' innovativeness.

Also, the willingness to cooperate is perceived as a personality trait that can influence innovation. Aerne argues that network actors work together to gain prestige [106]. Barroso-Castro and others have pointed out that directors’ ability to co-work on the board of listed companies is an important element of their internal social capital [88]. Castañer and Oliveira show that cooperation lies at the core of all inter-organisational activities [30]. Willingness to cooperate is also one of the critical factors in developing open innovation, both outside-in and inside-out [107]. As a result, another hypothesis was formulated.

H9 Willingness to cooperate positively affects SMEs' innovativeness.

Openness to experience (change) is a trait that refers to the extent to which people prefer novelty over convention. Being part of the Big Five personality traits, openness to experience measures curiosity and a willingness to search for new experiences and explore new ideas [108]. As Matz et al. suggest, this separates imaginative, creative people from down-to-earth and conventional people [109]. Mustafa et al. showed that openness to experience moderates the relationship between job satisfaction and innovative behaviour at work [95]. Saatci and Ovaci found that openness is relevant to individual innovation competencies [96]. Abdullah et al. showed that people with high openness to experience are more creative than others [46]. In turn, Ali's study found a positive influence of openness to experience on individual innovativeness and perceptions of satisfaction with life [91]. Accordingly, the following hypothesis was formulated.

H10 Openness to changes positively affects SMEs' innovativeness.

Risk-taking is a trait that implies consciously or unconsciously controlled behaviour with a sense of uncertainty about its outcome. It is also one of the critical characteristics of entrepreneurs who are perceived as more risk-taking than others [110]. Dyer et al. suggest that willingness to take risks distinguishes breakthrough innovators from others [105]. Many authors indicate risk-taking as an element of a company's innovative capacity, including Calantone et al. [111], Forsman [112] or Hull and Covin [99]. Also, a study by Martínez-Román et al. showed that risk-taking affects the innovativeness of Spanish SMEs [48]. On the other hand, it is also worth pointing out that excessive risk-taking in an enterprise may encounter resistance and fear of failure [52]. Consequently, the next hypothesis was proposed.

H11 Risk-taking positively affects SMEs' innovativeness.

Social empathy is also important from the perspective of internal cooperation and innovativeness. This is Segal's concept, which “*describes the insights one has about other people's lives that allow one to understand the circumstances and realities of other people's living situations*” [113]. Empathy for individuals is critical to personal growth [53]. People with empathy are more likely to be civic-minded and become responsible citizens [114]. Empathy has also been linked to emotional intelligence, in which it serves as a critical component [115]. Conversely, a lack of empathy strongly correlates with destructive tendencies [116], the worst of which may be sociopathic behaviours [117]. Social empathy is particularly visible in the social innovation perspective [[118], [119], [120]], understood as creating social innovation products to generate social value that responds to social challenges, including social and environmental problems [121,122]. As a result, another hypothesis was formulated.

H12 Social empathy positively affects SMEs' innovativeness.

**Fig. 1:**
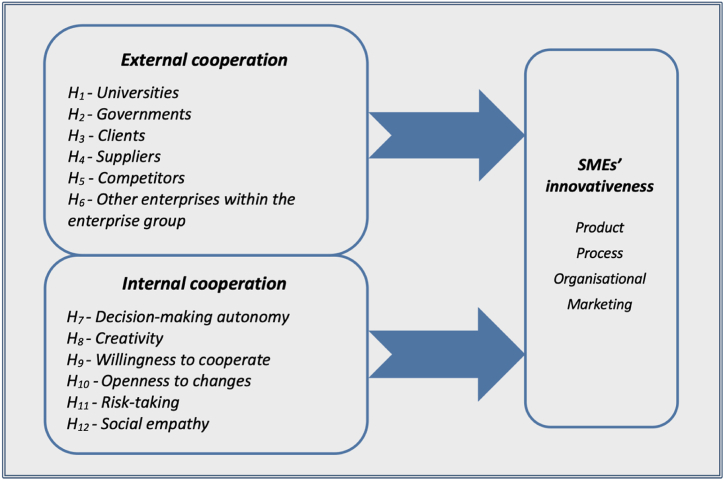
Conceptual model.

Consequently, the conceptual model presented in Fig. 1 was formulated.

## 3 Research methodology

### 3.1 Data collection and sample

The empirical data was obtained using the CAPI method (Computer-Assisted Personal Interviews) between June and September 2019. The SMEs participating in the study were based in the Kuyavian-Pomeranian Voivodeship in central-northern Poland. Based on the standards of the European Commission, enterprises with 10–49 employees were classified as small enterprises, while those with 50–249 employees were classified as medium-sized enterprises. The Statistical Office drew up the research sample based on Poland's National Official Register of Economy Entities (NOREE). The sample was stratified, with amounts for sectors according to the PKD 2007 (Code List of Classification of Business Activities in Poland), the subregion (NUTS 3) and county (NUTS 4), and was representative for the entire population of SMEs in the Kuyavian-Pomeranian Voivodeship (8177 SMEs in 2019), at a confidence level of 98% and an error of ± 3%. The original research sample included 3943 enterprises. The survey was addressed only to SME owners or managers. Ultimately, complete questionnaires were received from 1286 companies (79.1% small and 20.9% medium-sized) after incomplete responses were eliminated. This means a return rate of 32.6%.

The profile of the respondents is shown in Table 1. As can be seen, the largest group were men (68.5%), with non-economic education (70.6%), aged 40–49 (51.8%), with 4–10 years of work experience (42.1%) and owners of an SME (52.7%).

**Table 1:** Respondents' profile.

Characteristic	Sample %
*Gender*	
Female	31.5
Male	68.5
*Education*	
Economic	29.4
Non-economic	70.6
*Age (years)*	
up to 30	0.6
31–39	13.1
40–49	51.8
50–59	30.2
60 and above	4.4
*Work experience (years)*	
up to 3	4.3
4–10	42.1
11–20	41.2
21 and above	12.4
*Position*	
Owner	52.7
President/Vice-president	16.8
Manager	30.5

**Fig. 2:**
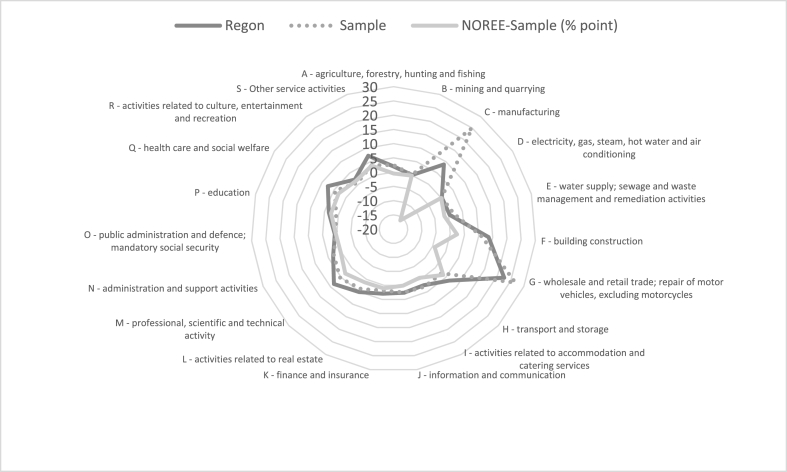
Structure of the sample.

As seen in Fig. 2, the survey sample represented all types of economic activity. Only two of these had a higher representation than in the REGON register: manufacturing (16.11%) and wholesale and retail trade (4.23%), while one had a lower representation, i.e., transport and storage (43.76%).

### 3.2 Variables

The dependent variables used were the indicators of SME innovations. SME owners and managers were asked whether they had introduced a product, process, organisational or marketing innovation in the previous three years (2016, 2017 and 2018). As a consequence, the following dummy variables were created.

• *Product innovation* (y_1_) – this variable takes the value one if the SME introduced a new or significantly improved product or service in the previous three years, and the value 0 otherwise;
• *Process innovation* (y_2_) – this variable takes the value one if the SME introduced a new or significantly improved production or delivery method in the previous three years, and the value 0 otherwise;
• *Organisational innovation* (y_3_) – this variable takes the value one if the SME introduced a new organisational method in the firm's business practices, workplace organisation or external relations in the previous three years, and the value 0 otherwise;
• *Marketing innovation* (y_4_) – this variable takes the value one if the SME introduced a new marketing method involving significant changes in product design or packaging, product placement, product promotion or pricing in the previous three years, and the value 0 otherwise.

The following independent variables, divided into three groups, were included in the study.

1. External cooperation: indicators of cooperation with triple helix entities, measured on a dichotomous scale (these variables take the value one if the SMEs cooperate with a specific entity, and the value 0 if otherwise):
  • Cooperation with a university (x_1_),
  • Cooperation with public administration – information support (x_2_),
  • Cooperation with public administration – financial support (x_3_),
  • Cooperation with clients (x_4_),
  • Cooperation with suppliers (x_5_),
  • Cooperation with competitors (x_6_),
  • Cooperation with other enterprises within the enterprise group (x_7_).
2. Internal cooperation: indicators of personality traits associated with cooperation, measured on an ordinal scale and taking values from 1 to 7, where 1 means complete disagreement and 7 represents full agreement:
  • *Decision-making autonomy* (x_8_) – Our employees have high decision-making autonomy,
  • *Creativity* (x_9_) – Our employees are creative,
  • *Willingness to cooperate* (x_10_) – Our employees cooperate willingly,
  • *Openness to changes* (x_11_) – Our employees are open to changes,
  • *Risk-taking* (x_12_) – Our employees take reasonable risks,
  • *Social empathy* (x_13_) – Our employees are involved in initiatives to solve social problems.
3. Control variables: the subject literature suggests that some control variables may affect SMEs' innovativeness. Earlier studies indicated, among other things, that the success of an enterprise may be a positive function of its age (experience) [32], that the size of an enterprise may be treated as a measure of its strength [32], and that the level of innovation may be influenced by the sector of activity [123]. Therefore, it was decided to use the following: enterprise age [32,124,125], enterprise size [32,[126], [127], [128]] and sector of economic activity [123,129,130]. Consequently, the following control variables were applied to the further analysis:
  • *Enterprise age* (x_14_) –SME age measured by the number of years since the business was founded – this variable was numerical, and a logarithm was applied to the calculations;
  • *Enterprise size* (x_15_) – SME size measured by the number of employees – this variable was numerical, and a logarithm was applied for the calculations;
  • *Sector of economic activity* – as a variable, this was initially collected on a nominal scale (three categories: *Industry*, *Services* and *Trade*). To enter them into the model and eliminate collinearity, it was necessary to create two binary variables: *Industry* and *Services*, and thus the third variable (*Trade*) became redundant. As a result, the following two variables were used:
    a. *Industry* (x_16_) – this variable takes the value one if the SME conducted industrial activities, and 0 otherwise;
    b. *Services* (x_17_) – this variable takes the value one if the SME conducted services activities, and 0 otherwise.

The statistical characteristics of all the analysed variables are presented in Table 2.

**Table 2:** Description of variables.

Variable	% - yes	Mean	S.E.	M	D	S.D.	SD2	Min.	Max.
y_1_	20.451	0.205	0.011	0	0	0.404	0.163	0	1
y_2_	17.481	0.174	0.011	0	0	0.379	0.144	0	1
y_3_	26.127	0.261	0.012	0	0	0.440	0.193	0	1
y_4_	21.461	0.215	0.011	0	0	0.411	0.169	0	1
x_1_	12.674	0.127	0.009	0	0	0.333	0.111	0	1
x_2_	13.297	0.133	0.009	0	0	0.340	0.115	0	1
x_3_	13.919	0.139	0.010	0	0	0.346	0.120	0	1
x_4_	56.609	0.809	0.013	1	1	0.393	0.155	0	1
x_5_	26.049	0.372	0.016	0	0	0.484	0.234	0	1
x_6_	1.244	0.018	0.004	0	0	0.132	0.017	0	1
x_7_	40.515	0.405	0.014	0	0	0.491	0.241	0	1
x_8_	–	4.336	0.038	4	4	1.362	1.856	1	7
x_9_	–	4.452	0.038	4	4	1.352	1.828	1	7
x_10_	–	4.684	0.038	5	4	1.348	1.818	1	7
x_11_	–	4.418	0.041	4	4	1.453	2.113	1	7
x_12_	–	4.217	0.042	4	4	1.493	2.229	1	7
x_13_	–	3.662	0.044	4	4	1.566	2.453	1	7
x_14_	–	2.871	0.017	2.996	3.367	0.609	0.371	0	5.017
x_15_	–	3.283	0.021	3.178	2.303	0.738	0.544	2.303	5.481
x_16_	24.183	0.242	0.012	0	0	0.428	0.183	0	1
x_17_	53.032	0.530	0.014	1	1	0.499	0.249	0	1

### 3.3 Method

The introduction by SMEs of a given type of innovation is not independent of the introduction of other innovations. Therefore, a multivariate probit model (MVP) was used considering the correlation of error terms [69,131]. MVP studies the effect of the independent variables on each introduced innovation type, while allowing for the correlation of unobserved and unmeasured factors (error terms). The correlation between the different kinds of innovations introduced may be the result of their interconnectedness (e.g., process innovation leads to product innovations) and is then positive, or, for example, due to SMEs’ limited resources (introduction of one type of innovation leads to the abandonment of others), and then it is negative. It is worth emphasising that if such a correlation occurs, the estimation of simple probit models may be biased and ineffective [[131], [132], [133]]. The proposed MVP model consists of four binary choice equations, namely the introduction of product, process, organisation and marketing innovation. Consequently, there are four dependent binary variables $y_{ij}$ for SME *i* and innovation *j*. This can be written as [131]:

$$\begin{array}{cc} y_{ijm}^{*}=X_{ijm}^{\prime }\beta_{m}+\varepsilon_{ijm} & m=1,2,3,4 \end{array}$$

$$y_{ijm}=\left(\begin{array}{l} 1\;if\;y_{ijm}^{*}>0\\ 0\;otherwise \end{array}\right)\text{,}$$

where $y_{ijm}^{*}$ is a latent variable that captures the degree to which SMEs view innovation m as worth introducing. This latent variable is assumed to be a linear combination of observed external and internal cooperation characteristics $X_{ijm}^{*}$, and unobserved characteristics captured by the stochastic error term $\varepsilon_{ijm}$. The vector of the parameters to be estimated is denoted by $\beta_{m}$. Considering the latent nature of $y_{ijm}^{*}$, the estimation is based on the observable binary $y_{ijm}$, indicating whether an SME introduced a particular innovation in the previous three years (2016, 2017 and 2018).

The error terms $\varepsilon_{ijm}$ (m=1,2,3,4) have normal multivariate distribution, each with means of 0 and a variance-covariance matrix V, where V has 1 on the leading diagonal, and correlations $p_{jk}=p_{kj}$ as off-diagonal elements [131].

To quantify the marginal effects of the independent variables, the probability of each innovation introduction, written as:

$$\mathrm{Pr}\left(y_{ijm}=1\right)=\Phi \left(y_{ijm}^{*}\right)\text{,}$$

can be differentiated, where Φ(.) is the univariate standard normal cumulative distribution function [133].

To estimate all the models, the simulated maximum likelihood estimation method [134] and STATA.16.1 software were used.

## 4 Results and discussion

### 4.1 Estimation results

The final results of the estimations are shown in Table 3. Based on the likelihood ratio test, the null hypothesis of zero correlation between the error components should be rejected (P < 0.0000). This means that MVP is preferred over single-equation probit models.

**Table 3:** Multivariate probit model results.

Variable	Model 1 (y_1_)			Model 2 (y_2_)			Model 3 (y_3_)			Model 4 (y_4_)		
	*β*	S.E.	dF/dx	*β*	S.E.	dF/dx	*β*	S.E.	dF/dx	*β*	S.E.	dF/dx
x_1_	0.636**	0.132	0.007**	0.280*	0.132	0.005*	0.206	0.126	0.005	0.380**	0.128	0.028**
x_2_	0.219	0.146	0.003	0.129	0.144	0.002	0.245	0.135	0.006	−0.144	0.146	−0.011
x_3_	0.365**	0.135	0.004**	0.477**	0.131	0.008**	0.406**	0.127	0.010**	0.295*	0.130	0.022*
x_4_	0.307**	0.110	0.004**	0.177*	0.107	0.003*	0.323**	0.097	0.008**	0.246*	0.099	0.018*
x_5_	0.191	0.103	0.002	−0.055	0.102	−0.001	−0.079	0.093	−0.002	0.428**	0.093	0.032**
x_6_	0.224	0.342	0.003	0.386	0.319	0.007	0.674	0.354	0.016	0.096	0.318	0.007
x_7_	0.119	0.109	0.001	0.176	0.108	0.003	0.299**	0.096	0.007**	0.110	0.099	0.008
x_8_	0.074	0.045	0.001	0.030	0.043	0.001	0.057	0.038	0.001	0.065	0.039	0.005
x_9_	0.185**	0.058	0.002**	0.168**	0.055	0.003**	0.061	0.046	0.001	0.124*	0.049	0.009*
x_10_	−0.027	0.059	0.000	0.014	0.056	0.000	0.106*	0.048	0.003*	0.007	0.050	0.001
x_11_	0.121*	0.054	0.001*	0.036	0.052	0.001	−0.033	0.046	−0.001	−0.082	0.048	−0.006
x_12_	−0.015	0.040	0.000	−0.019	0.038	0.000	0.049	0.035	0.001	0.113**	0.036	0.008**
x_13_	0.172**	0.031	0.002**	0.155**	0.031	0.003**	0.007	0.028	0.000	0.113**	0.029	0.008**
x_14_	0.013	0.076	0.000	−0.021	0.074	0.000	0.003	0.068	0.000	−0.060	0.069	−0.004
x_15_	−0.047	0.062	−0.001	0.090	0.061	0.002	0.154**	0.056	0.004**	−0.053	0.058	−0.004
x_16_	−0.027	0.129	0.000	0.136	0.130	0.002	−0.131	0.117	−0.003	−0.035	0.117	−0.003
x_17_	−0.457**	0.115	−0.005**	−0.130	0.114	−0.002	−0.230*	0.100	−0.006*	−0.533**	0.102	−0.040**
Constant	−3.238**	0.362	0.007**	−3.161**	0.348	0.005**	−2.602**	0.313	0.005**	−2.023**	0.317	0.028**
rho21	0.771**	0.029										
rho31	0.589**	0.041										
rho41	0.661**	0.036										
rho32	0.543**	0.042										
rho42	0.669**	0.035										
rho43	0.621**	0.036										
Log likelihood		−1806.539										
Wald chi^2^ (68)		489.49										
Prob > chi^2^		0.0000										

* p-Value ≤0.05, ** p-Value ≤0.01. *Notes: N* = 1286; Likelihood ratio test of rho21 = rho31 = rho41 = rho32 = rho42 = rho43 = 0: chi^2^ (6) = 667.683, Prob > chi^2^ = 0.0000.

Table 3 shows the estimates of the coefficients and the marginal effects. It should be clarified that the marginal effects indicate the strength of the relationship between the accompanying variables and the implementation of innovations by SMEs. For the dummy variables, the marginal effect refers to the change of the variable from 0 to 1.

The significance and high values of rho21 (0.771) indicate a high positive correlation between SMEs' introduction of product and process innovations. There is also a stronger correlation between product and marketing innovations (rho41) than between product and organisational innovation (rho31). In the case of process innovations, these are more strongly correlated with marketing innovations (rho42) than organisational innovations (rho32). The correlation between organisational and marketing innovations is also high (rho43).

The estimated parameters, which were statistically significant, took only positive values (except for the control variables), which means that the impact of the independent variables on the dependent variable increases the probability of an SME introducing a product, process, organisational or marketing innovation.

### 4.2 Discussion

This paper's main objective was to analyse the external and internal cooperation determinants of SME innovations. The proposed conceptual model divided the possible determinants into two groups: external cooperation (the perspective of the triple helix) and internal cooperation (selected employee personality traits).

As seen in Table 3, only two variables are common, statistically significant determinants explaining all types of SME innovations: x_3_ - cooperation with public administration – financial support, and x_4_ - cooperation with clients. This means that only in the case of hypotheses H_2_ and H_3_ is there no reason to reject them (p ≤ 0.05).

However, it is worth noting that there is a difference between the other determinants within each model. For product innovation, x_1_, x_9_, x_11_ and x_13_ are also crucial determinants. Meanwhile, x_1_, x_9_ and x_13_ are determinants in the case of process innovation. In the next model – for organisational innovation – the determinants are x_7_ and x_10_, while in the marketing innovations model, the remaining determinants are x_1_, x_5_, x_9_, x_11_, x_12_ and x_13_. The results of the hypothesis testing are summarised in Table 4.

**Table 4:** Hypothesis testing results.

Hypothesis	Innovation			
	Product	Process	Organisational	Marketing
H_1_	Supported	Supported	Rejected	Supported
H_2_	Supported	Supported	Supported	Supported
H_3_	Supported	Supported	Supported	Supported
H_4_	Rejected	Rejected	Rejected	Supported
H_5_	Rejected	Rejected	Rejected	Rejected
H_6_	Rejected	Rejected	Supported	Rejected
H_7_	Rejected	Rejected	Rejected	Rejected
H_8_	Supported	Supported	Rejected	Supported
H_9_	Rejected	Rejected	Supported	Rejected
H_10_	Supported	Rejected	Rejected	Rejected
H_11_	Rejected	Rejected	Rejected	Supported
H_12_	Supported	Supported	Rejected	Supported

The results suggest that SMEs which cooperate with a university have a greater probability of introducing product (0.7%), process (0.5%) and marketing innovations (2.8%) than other SMEs. This result confirms previous studies, e.g. Prokop and Stejskal [70] and Prokop [64]. It may also suggest that the lack of trust and limited cooperation with universities in CEE countries, as indicated by Prokop [64], has changed in the case of Poland. It is worth noting that while in the case of product and process innovations, this influence was indicated in earlier studies [6,69], in the case of marketing innovations, it was not. Due to their character, according to Oslo Manual, these comprise the implementation of a new marketing method involving significant changes in product design or packaging, product placement, product promotion or pricing [54] - and may indicate SMEs' cooperation with universities in the area of marketing and management.

The cooperation of SMEs with public administration was analysed from two perspectives - information and financial support. In the case of the former, no correlation was observed concerning innovations introduced by SMEs. This contradicts previous studies, which suggest such an impact on product and process innovations [6,48] and the diffusion of innovations [72]. In turn, in the case of financial support, the MVP results showed a positive impact on the introduction of innovations. SMEs cooperating in this way are more likely to introduce product (0.4%), process (0.8%), organisational (1%) and marketing (2.2%) innovations.

In the field of cooperation between firms, SMEs’ cooperation with clients has a significant influence on the probability of introducing innovation. By cooperating with clients, SMEs are 0.4% more likely to introduce product innovation and 0.3% more likely to implement process innovation, with 0.8% for organisational innovation and 1.8% for marketing innovation. This indicates the strong relations between SMEs and clients and the implementation of customer-orientated innovations. This result confirms earlier studies [48,73,82,135].

It is interesting that cooperation with suppliers positively impacts SMEs' innovativeness only in the scope of marketing innovations. SMEs who cooperate with suppliers are 3.2% more likely to engage in the introduction of this kind of innovation. The lack of impact on product and process innovations is surprising. As was suggested in previous research, such cooperation can bring several benefits, e.g. improving the quality of products or reducing costs [48,73].

The results also did not show an impact of cooperation with competitors on the innovativeness of the SMEs studied. This is an interesting and surprising result, especially in the context of product and process innovations, where such cooperation can bring several benefits, such as access to knowledge and technological skills, or higher efficiency in the use of resources [6,32,49,81]. On the other hand, there are also results from studies by Giovannetti and Piga which suggest that active cooperation in innovation activities between competitors lowers their innovation indicators and intermediate productivity [75].

In turn, cooperation with other enterprises within a group only increases the likelihood of introducing organisational innovations, by 0.7%. This result partially confirms Tether's findings [82]. It could be assumed that belonging to a group of enterprises would result in a more effective flow of information between group members, increasing their absorption capacity and resulting in more significant innovation in products and technologies.

Analysing the results from the perspective of internal cooperation - based on selected personality traits, understood as an essential element of internal cooperation that may influence SME innovations - it transpires that none of the possible employee personality traits were found to have a statistically significant impact on any type of SME innovation. Generally, considerable variation was observed in the field of internal cooperation.

Decision-making autonomy, as a personality trait affecting internal cooperation, did not influence the introduction of innovations by the SMEs studied. This result is surprising in the context of previous studies, e.g. Martinez-Roman et al. [48,49], Russell [98], Yam et al. [100] or Hull and Covin [99], which suggested that the autonomy of decision-making is of great importance for the innovative activity of enterprises.

In turn, in the case of creativity, SMEs whose employees are creative have a greater probability of introducing product (by 0.2%), process (0.3%) and marketing innovations (0.9%). This result is not surprising. According to Runco, creativity is an important part of cognitive, social and emotional activity [101]. Creativity has been indicated in many studies as a personality trait influencing a firm's innovativeness [29,50,52,102]. What is puzzling, however, is the lack of impact in the case of organisational innovation. After all, it would seem to be critical here as well.

SMEs whose employees cooperate willingly are 0.3% more likely to introduce only organisational innovation. This result is also surprising. Aerne suggested that network actors work together to gain prestige [106]. In contrast, Barroso-Castro and others believe that the ability to cooperate is an essential element of their internal social capital [88]. As a personality trait, willingness to cooperate was indicated in other research as a factor influencing the implementation of product and process innovation, e.g. Mir et al. [31] and Romijn and Albaladejo [51]. The result observed in this study is inconsistent with this.

Further, openness to change, as a personality trait connected with internal cooperation, positively affects the probability of innovation introduction. This was observed in one case. Those SMEs whose employees are open to changes have a greater likelihood of introducing product innovations by 0.1%. It is puzzling that openness to experience (change), one of the fundamental personality traits of the Big Five model [108], affects only one type of innovation. After all, earlier studies showed the relationship between openness and innovative behaviour at work [95], individual innovative competencies [96] and personal innovativeness [91].

In turn, risk-taking turned out to be statistically significant only in the case of marketing innovations. Consequently, SMEs whose employees take reasonable risks have a greater 0.8% probability of introducing such innovations. This is also quite an exciting result, especially if it is viewed from the perspective of earlier research suggesting that risk-taking is a feature that distinguishes breakthrough innovators from others [105], and is one of the essential elements of a company's innovative capability [99,111,112].

An interesting result is also connected with the personality trait of social empathy. SMEs whose employees are involved in initiatives solving social problems are 0.2% more likely to engage in the introduction of product innovation, 0.3% in process innovation and 0.8% in marketing innovation. This shows that social empathy can influence the creation of products that can act as social innovations [121,122].

It is worth mentioning that two control variables also have a statistically significant influence on the introduction of innovations by SMEs. As observed, having additional SME employees increases the probability of introducing organisational innovation by 0.4%. In turn, in the case of the sector of economic activity, the results suggest that service SMEs have a lower probability of introducing product (by 0.5%), organisational (by 0.6%), and marketing innovations (by 4%) than trade SMEs.

## 5 Conclusions

In this paper, the external and internal cooperation determinants of four innovation types - product, process, organisational and marketing - were studied from the perspective of SMEs in the Kuyavian-Pomeranian voivodeship, a region in central-northern Poland.

From the theoretical perspective, considering the dual nature of cooperation, it was deemed necessary to divide the cooperation determinants into two groups: external (with triple helix entities) and internal (employee personality traits). Additionally, three control variables were considered: company age and size, and sector of economic activity.

The proposed model is original in the following aspects: (a) it explains the four types of innovations, (b) it includes a broad spectrum of factors specific to external and internal cooperation that have not been sufficiently researched so far (especially in the field of employee personality traits), and (c) it provides the possibility to take a new look at SMEs’ innovativeness, and predicts some possible new aspects of their functioning that are important from a management and support policy point of view.

The results indicate that only two factors directly connected with the triple helix are common and significant determinants that explain all SME innovations. These are cooperation with public administration in the field of financial support, and cooperation with clients.

Significant variation was observed regarding personality traits as an essential element of internal cooperation that may influence SMEs’ innovations. The positive impact on the probability of implementing three of the four types of innovation was observed for two personality traits, namely creativity and social empathy.

It is worth emphasising that this research has direct managerial and policy implications.

Firstly, cooperation with public administration with regard to financial support results in greater SME innovations. From the perspective of aspiring to increase the regional level of innovativeness, it is worth attempting to expand and intensify these forms of triple helix cooperation.

Secondly, cooperation with clients significantly influences the probability of introducing innovation. This indicates the strong relations between SMEs and clients and the implementation of customer-orientated innovations. This kind of cooperation should be stimulated and supported.

Thirdly, employees' creativity should be particularly encouraged from a managerial perspective as it stimulates internal cooperation and contributes to the improvement of innovation.

Fourthly, an important role is demonstrated of SMEs' employee's engagement in initiatives solving social problems (social empathy). So, if the aim is to increase the innovativeness of a given region, SMEs should be encouraged to become more involved in local social initiatives. Possible activities may include practical policies, educational programmes, and financial support for local social initiatives.

This research has some limitations which may give rise to possible future research.

Firstly, the limitations result from the proposed research model, based on cooperation with triple helix units and selected employee personality traits. Future research could include a more elaborate concept of external collaboration actors (e.g. resulting from national and regional eco-innovation systems), as well as the personality traits influencing internal collaboration (e.g. based on the Big Five model).

Secondly, the study did not include micro-enterprises and the self-employed. Future research could be narrowed down to microenterprises or the self-employed either combined or separately, which could bring valuable insights into the dual nature of the cooperation determinants of innovativeness.

Thirdly, the research results may be applied only to the Kuyavian-Pomeranian region and similar areas in Poland.

Lastly, the research shows the external and internal cooperation determinants of SME innovation at a specific moment. From this perspective, it would be worth carrying out a longitudinal study in the future.

## Author contribution statement

Maciej Zastempowski: Conceived and designed the analysis; Analysed and interpreted the data; Contributed analysis tools; Wrote the paper.

## Data availability statement

Data will be made available on request.

## Additional information

Supplementary content related to this article has been publish online at [URL].

## Declaration of competing interest

The author declares that he has no known competing financial interests or personal relationships that could have appeared to influence the work reported in this paper.
